# Alteration in molecular structure of alkali activated slag with various water to binder ratios under accelerated carbonation

**DOI:** 10.1038/s41598-022-09491-4

**Published:** 2022-04-01

**Authors:** Thi Nhan Nguyen, Quoc Tri Phung, Ziyou Yu, Lander Frederickx, Diederik Jacques, Dimitrios Sakellariou, Alexandre Dauzeres, Jan Elsen, Yiannis Pontikes

**Affiliations:** 1 Institute for Environment, Health, and Safety, Belgian Nuclear Research Centre (SCK CEN), 2400 Mol, Belgium; 2 cMACS, Department of Microbial and Molecular Systems (M2S), KU Leuven, 3001 Leuven, Belgium; 3 Institute of Radiation Protection and Nuclear Safety (IRSN), 92260 Fontenay-aux-Roses, France; 4 Department of Earth and Environmental Sciences, KU Leuven, 3001 Leuven, Belgium; 5 Department of Materials Engineering, KU Leuven, 3001 Leuven, Belgium

**Keywords:** Chemical engineering, Civil engineering, Composites, Organic-inorganic nanostructures, Solid-state chemistry, Geochemistry, Characterization and analytical techniques

## Abstract

Carbonation of alkali activated materials is one of the main deteriorations affecting their durability. However, current understanding of the structural alteration of these materials exposed to an environment inducing carbonation at the nano/micro scale remains limited. This study examined the evolution of phase assemblages of alkali activated slag mortars subjected to accelerated carbonation (1% CO_2_, 60% relative humidity, up to 28 day carbonation) using XRD, FTIR and ^29^Si, ^27^Al, and ^23^Na MAS NMR. Samples with three water to binder (w/b) ratios (0.35, 0.45, and 0.55) were investigated. The results show that the phase assemblages mainly consisted of C-A-S-H, a disordered remnant aluminosilicate binder, and a minor hydrotalcite as a secondary product. Upon carbonation, calcium carbonate is mainly formed as the vaterite polymorph, while no sodium carbonate is found after carbonation as commonly reported. Sodium acts primarily as a charge balancing ion without producing sodium carbonate as a final carbonation product in the 28-day carbonated materials. The C-A-S-H structure becomes more cross-linked due to the decalcification of this phase as evidenced by the appearance of Q^4^ groups, which replace the Q^1^ and Q^2^ groups as observed in the ^29^Si MAS NMR spectra, and the dominance of Al(IV) in ^27^Al MAS NMR. Especially, unlike cementitious materials, the influence of w/b ratio on the crystalline phase formation and structure of C-A-S-H in the alkali activated mortars before and after carbonation is limited.

## Introduction

Alkali activated materials (AAMs) produced from aluminosilicates and alkaline activators have attracted a lot of attention over the past decades owing to their environmental and economic benefits compared to ordinary Portland cement (OPC) based materials^1–3^. Among them, alkali activated slag (AAS) formed from granulated blast furnace slag (GBFS), one of the major aluminosilicate sources, and alkaline solutions has been studying intensively^4–6^. AAMs have demonstrated satisfactory performances in several applications^7^. The (long-term) durability of these materials is, however, still uncertain. There is a need to further investigate the performance of AAMs under various environmental conditions to assess the durability of these materials and to make them more acceptable in various engineering applications^8^.

Carbonation is one of the crucial durability issues of reinforced cement and concrete as the process reduces the pH in the pore solution of materials, leading to the corrosion of reinforcing steel bars due to the destruction of its passivating layer^9,10^. Alkali activated materials are also expected to be carbonated during their service life. However, the carbonation mechanism and its effects on material alteration may be different from those of OPC systems due to the differences in chemistry and pore structure. Few studies on the carbonation of AAMs have been carried out in recent years to understand the carbonation process and its influence on the performance of the AAMs. For example, several studies^6,11^ have shown that AAMs are more vulnerable to carbonation than OPC-based materials. Because of the absence of Ca(OH)_2_ that buffers the solution to a high pH and then delays the decalcification of calcium silicate hydrate (C-S-H)^12^ in OPC systems, decalcification of aluminosilicate phase C-(N)-A-S-H occurs faster. Bernal et al*.* demonstrated that carbonation of AAS occurred in two stages^13^: (i) carbonation of the pore solution resulting in a reduction of pH and the Na-rich carbonates precipitation eventually; (ii) decalcification of C-(N)-A-S-H phase and secondary phases leading to the formation of CaCO_3_ and probably carbonated hydrotalcite.

Despite a relatively high number of studies^14–17^ on the carbonation of AAMs, a systematic approach is still missing to investigate different factors influencing the carbonation process such as precursor type^18–20^, alkaline activator^21,22^, CO_2_ concentration^23^, curing temperature^17^, and relative humidity. Among these, CO_2_ concentration can be considered to play a key role in influencing the process and carbonation products^24^. Bernal et al*.* reported that the carbonation rate in pore solution and aluminosilicate gel of GBFS/metakaolin based geopolymers at higher CO_2_ concentration (3%) seemed significantly different compared to that at 1% CO_2_^18^. They also highlighted that bicarbonate products were favored over carbonates at higher CO_2_ concentration. This is in line with the study of Pouhet et al*.*^19^ that sodium carbonate was formed in the pore solution of metakaolin-based geopolymer under natural carbonation, while sodium bicarbonate was observed at 50% CO_2_. In addtion, Shi et al*.* found that the natural carbonation rate of AAS concrete was only approximately 1 mm/year, while its carbonation depth under 7% CO_2_ ranged from 13 to 25 mm after just 10 days^7^. Thus, accelerated carbonation at high CO_2_ concentration (> 1%) was considered to be not representative of the behavior of AAS under atmospheric CO_2_ conditions in real service life, because of changes in the carbonate phase equilibrium in pore solution with an increase in CO_2_ concentration^19^.

The phases and chemistry of gels produced in AASs are also controlled by the chemistry of the raw materials^25,26^ and the utilized activators^5,27^, consequently, carbonation of AASs may depend on these factors. Regarding the slag chemistry described in the quaternary oxide system CaO–MgO–Al_2_O_3_–SiO_2_, the predominant components CaO and SiO_2_ control the Ca/Si ratio of the C-A-S-H phase. Under carbonation exposure, this phase leaves a cross-linked and remnant silicate phase resulting from the decalcification of the C-A-S-H and carbonate precipitate^28^. Haha et al.^29^ identified that an increase in Mg content from 7.7 to 13.2% leads to additional formation of double-layered hydroxides of hydrotalcite which adsorb CO_2_ and thereby reduce the carbonation of C-A-S-H^30^. An increase in the Al_2_O_3_ content to approximately 16% combined with a low MgO content (< 5%) leads to the formation of zeolites instead of hydrotalcite^31^. Aside from the slag precursor, the activator is also a crucial parameter in the carbonation process of AASs. An increase in the Na_2_O concentration of activators enriches Na^+^ in the C-A-S-H network and forms C-N-A-S-H, thereby reducing the susceptibility to carbonation^32^.

The insight into the influence of the water to binder (w/b) ratio, an important factor in the carbonation of OPC^33^ and in determining the performance of AAMs in general and carbonated, is still limited. The w/b ratio is expected to change the rate of the precursor dissolution and the geopolymerization of AAMs^34^, which then defines the microstructure of polymerized products. Provis et al*.* pointed out that the water content changes the alkali activator concentration that influences the reaction rate and also the structure of reaction products^35^. Mobili et al*.* reported that lower w/b ratios produce AASs with lower total porosities and higher densities^36^. These authors also indicated a linear relation between the w/b ratio and an AAS’s density, demonstrating that an increase of free water results in a more porous matrix. Consequently, AAMs formulated with different w/b ratios can develop different transport properties^37^, which then affect the diffusion of CO_2_ and thereby the carbonation process. Recently, Zhang et al*.* studied the effect of the w/b ratio on the carbonation of alkali activated slag/fly ash and found that a decrease in the w/b ratio can reduce the formation rate of Ca-carbonates in alkali activated slag-rich blends, but the crystallinity of these Ca-carbonates is higher^38^. However, the effect of the w/b ratio on the alteration of the gel structure after carbonation is not discussed in detail in their study.

This study addresses the gap in understanding of the alteration of the nano/micro structure of AASs under accelerated carbonation conditions (1% CO_2_, 60% RH) and the influence of the w/b ratio on the phase evolution of uncarbonated and carbonated materials. Multiple mineralogical characterization techniques have been used including X-ray diffraction (XRD), Fourier transform infrared spectroscopy (FTIR), and high resolution solid state ^29^Si, ^27^Al and ^23^Na magic angle spinning (MAS) nuclear magnetic resonance (NMR) spectroscopy to comprehensively assess the changes in phase assemblages and C-A-S-H structure of AASs. By combining strong experimental evidence and geochemical modelling, a new mechanism for the carbonation process is proposed to better understand the carbonation mechanism of AASs.

## Materials and methods

### Materials and mixing procedure

The granulated blast furnace slag was supplied by Ecocem Benelux with a chemical composition determined by X-ray fluorescence (XRF)^39^ listed in Table 1. Solid NaOH pellets (99% purity, VWR Chemicals) were dissolved in deionized water to obtain a solution of 5 mol/l, which was then mixed with a commercial sodium silicate solution from VWR Chemicals (consisting of 8.97% Na_2_O, 26.78% SiO_2_, and 64.25% H_2_O) to form the alkali activating solution. The activator dosage followed the recommended one reported in the RILEM TC 247-DTA round robin test (i.e. 4 g NaOH/100 g GBFS and 2.69 g Na_2_O·2SiO_2_/100 g GBFS)^40^. The activator was kept in a fume hood for approximately 24 h to cool to room temperature before use. Tap water was added to the activating solution to reach the target w/b ratios of 0.35, 0.45 and 0.55. River sand was used as a fine aggregate with a maximum particle size of 2 mm and a density of 2.67 g/cm^3^. The volume fraction (relative to total volume of the mortar) of the river sand was fixed at 0.2 (relevant for waste encapsulation context). The mix designs are given in Table 2.

**Table 1:** Chemical compositions of GBFS (wt%).

Oxides	SiO_2_	Al_2_O_3_	Fe_2_O_3_	CaO	MgO	K_2_O	Na_2_O	TiO_2_	SO_3_	L.O.I
GBFS	32.4	11.1	0.60	43.40	7.77	0.53	0.27	1.01	2.41	0.51

**Table 2:** Mix design for AAS mortars.

Mortars	w/b	SiO_2_/Al_2_O_3_	SiO_2_/Na_2_O	H_2_O/Na_2_O
AAS 035	0.35	5.23	8.79	32.02
AAS 045	0.45	5.23	8.79	41.17
AAS 055	0.55	5.23	8.79	50.32

The activating solution and additional water were mixed for 1 min in a mixing bowl, and then GBFS was added and mixed for 2 min using a rotary mixer at a low speed. Afterwards, sand was poured into the mixture and the mixing was continued for 2 min. The mixer was stopped to scrape the mortar of the wall of the bowl, and was finally mixed at high speed for another 2 min. The mortar was then cast in 40 × 40 × 160 mm moulds and kept at room temperature under sealed conditions for 24 h. Finally, samples were demoulded and placed in a curing cabinet at 20 °C and a relative humidity exceeding 95% RH for 27 days.

### Accelerated carbonation test

**Figure 1:**
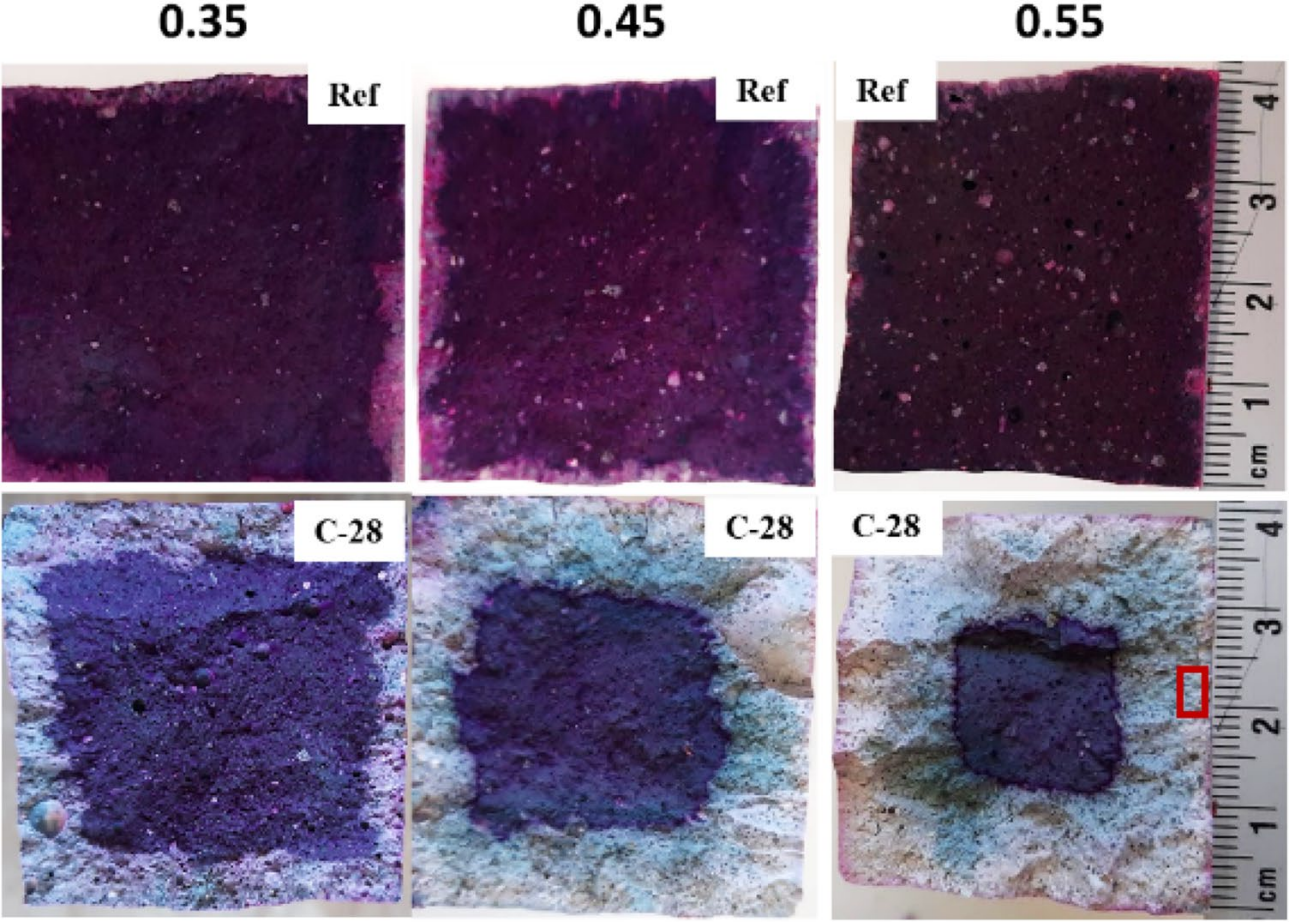
Example of carbonation depth determined by phenolphthalein spraying of sample after preconditioning (Ref) and 28 day carbonated (C-28) samples. Pink color indicates the uncarbonated zone. The red rectangular indicates the location to take sub-samples for characterization.

After 28 days of curing, the samples were conditioned in a 20 °C climate chamber until the mass difference between two measurements within 24 h was less than 0.2% to reach a target RH of 60% (around 4 weeks). Afterwards, the samples were placed in the carbonation chamber for accelerated carbonation tests. The carbonation conditions were set at temperature of 20 °C, RH of 60%, and CO_2_ concentration of 1% following EN standard 13295^41^. The carbonated zone was determined by phenolphthalein indicator on samples subjected to accelerated carbonation after 7, 14, and 28 days. Figure 1 shows an example for carbonation depths determined by the phenolphthalein spraying method for carbonated AAS with 3 w/b ratios of 0.35, 0.45, and 0.55 after 28 days of carbonation. Sub-samples were then taken from the carbonated zone within 3 mm from the reactive surface for characterization (Fig. 1). Also, sub-samples were taken from the 28 day cured reference samples for characterization.

### X-ray diffraction (XRD)

After freeze drying, samples for quantitative XRD were crushed in a mortar, sieved to a size below 63 µm and mixed with 10% of the internal standard zincite (ZnO)^42^. The powders were then measured as quickly as possible (within a few hours) to prevent atmospheric carbonation. Measurements were performed with a Bruker D8 instrument equipped with a Cu Kα source, a beam knife, a Ni filter and automated divergence slits. Operational settings were set at 40 kV and 40 mA and measurements were performed between 5° and 60° 2θ at a step size of 0.02° 2θ. Quantification was subsequently performed using Rietveld refinement with the Profex software^43^, which is a graphical user interface of the BGMN code.

### Fourier-transform infrared spectroscopy (FTIR) measurements

The freeze-dried powder samples were homogenized with KBr in a 0.05 sample to KBr ratio, after which the mixtures were measured by diffuse reflectance infrared Fourier transform (DRIFT) spectroscopy on a Bruker Tensor II spectrometer. Measurements were carried out in the wavelength range of 4000–400 cm^−1^ at a resolution of 4 cm^−1^ at a total of 50 scans per measurement.

### Solid-state ^29^Si, ^27^Al and ^23^Na MAS NMR

High resolution solid-state ^29^Si, ^27^Al and ^23^Na magic angle spinning (MAS) nuclear magnetic resonance (NMR) spectra were acquired at 25 °C on a Varian Inova 500 MHz wide more NMR spectrometer (${B}_{0}=11.7 \,T$) using a single pulse sequence. The resonance frequency of ^29^Si, ^27^Al and ^23^Na were at 99.3 MHz, 130.23 MHz and 132.21 MHz, respectively. The ^29^Si MAS NMR spectra were obtained using a Chemagnetics 7.5 mm double air bearing CPMAS probe and rotors spinning at 6 kHz with a 90° pulse width of 3.42 μs, a recycle delay of 30 s and 1680 scans. High power ^1^H decoupling was applied during the acquisition. The ^27^Al and ^23^Na MAS NMR spectra were measured using a Chemagnetics 2.5 mm double air bearing CPMAS probe and rotors spinning at 15 kHz with a pulse length of 2–2.2 μs, a recycle delay of 0.5 s and 1024 up to 2048 scans. All the spectra were measured in ZrO_2_ rotors to eliminate the influence of any Si, Al background. The ^29^Si MAS NMR spectra were referenced to an external standard sample of tetrakis(trimethylsilyl)silane (TKS). The peak position of ^27^Al and ^23^Na MAS NMR spectra were referenced to 1 M Al(NO_3_)_3_ and 1 M NaCl solution, respectively.

Deconvolution of ^29^Si MAS NMR spectra was performed with the FitYK software^44^ using Lorentzian functions for the crystalline peaks and Gaussian functions for the broaden peaks. The full width at half height of each peak was constrained within 10 ppm^28,45^. The minimum number of fitting peaks was chosen to describe the spectra. The peak positions were referenced from literature^28,46–48^.

## Results and discussion

### X-ray diffraction

**Figure 2:**
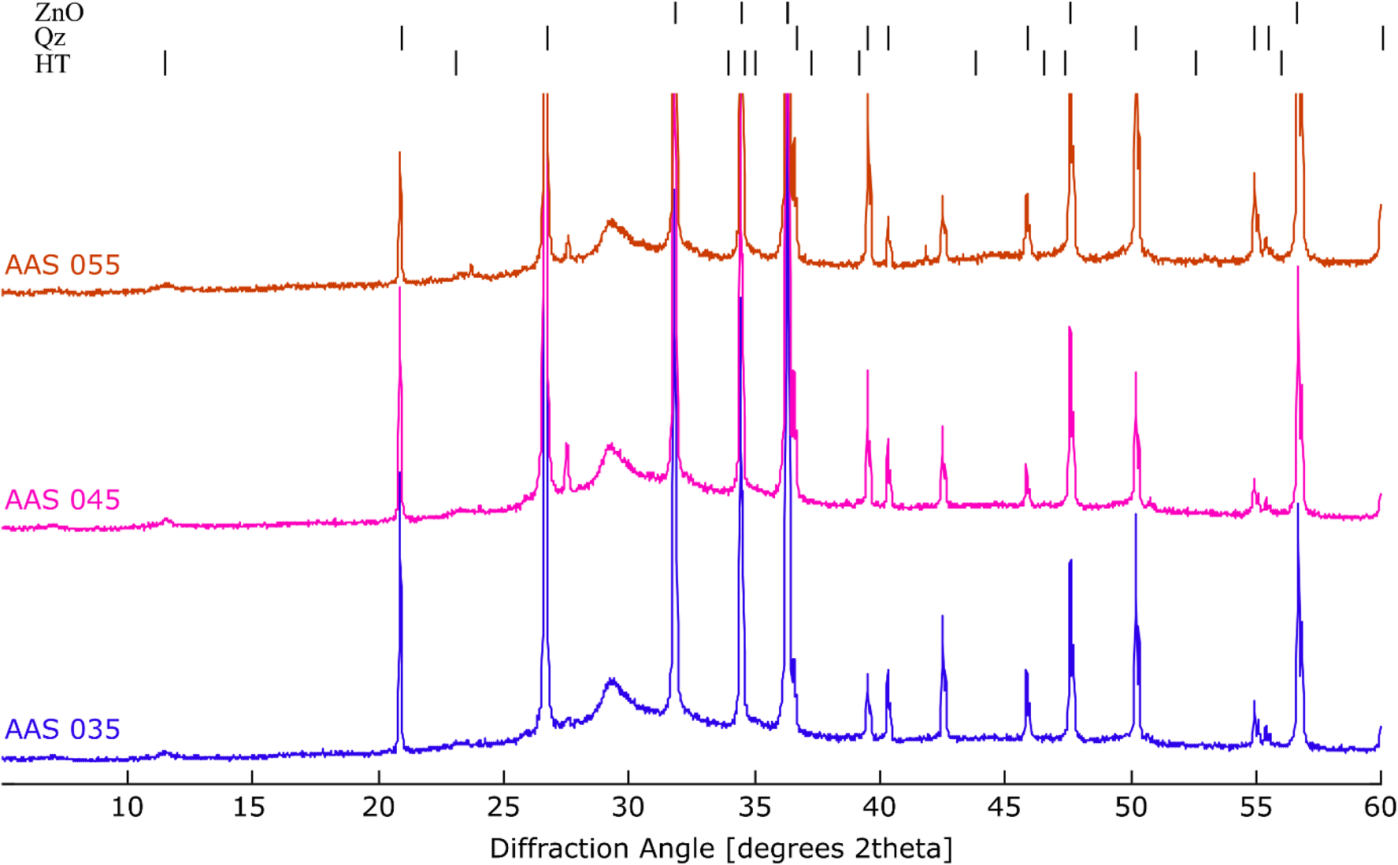
XRD diffractograms of the reference specimens of each water to binder ratio: *ZnO* zincite internal standard, *Qz* quartz, *HT* hydrotalcite.

The XRD diffractograms (Fig. 2) of the uncarbonated alkali-activated slag specimens are dominated by a mixture of amorphous phases with a broad peak at 29.16° 2θ, which can be related to a combination of unreacted slag particles and newly formed C-A-S-H phase^49^. The main crystalline phase is quartz, which is associated with the aggregate, as well as smaller amounts of K-feldspar. An AFm-phase with a peak at 11.53° 2θ, best modeled by hydrotalcite, is also present in small amounts (1%). Note that with the presence of aggregate in the samples, more attention is needed to interpret the XRD data because aggregate particles are typically not homogeneously distributed in the sample at small scale (few grams for XRD measurement). To eliminate the effect of the heterogeneous distribution of aggregate, quartz and K-feldspar were removed from the quantified composition, after which the rest of the phases were renormalized to 100%. In that way, the composition reported in Table 3 could be interpreted as that of the paste (excluding aggregates). The proportion of amorphous phase at paste level, calculated by the internal standard method, is quite similar (97–98%) for all uncarbonated samples with various w/b ratios as shown in Table 3.

**Table 3:** Quantitative phase analysis for reference and carbonated AAS specimens at three w/b ratios.

Phases	AAS 035		AAS 045				AAS 055	
	Ref	28D	Ref	7D	14D	28D	Ref	28D
Amorphous	98.8	97.6	97.5	92.9	92.4	87.4	98.8	85.0
Hydrotalcite	1.2	1.2	2.5	1.2	1.3	1.2	1.2	1.2
Vaterite	0.0	1.2	0.0	6.0	6.3	11.4	0.0	13.7
Total	100	100	100	100	100	100	100	100

*Ref* uncarbonated samples, *7D, 14D, 28D* 7-day, 14-day and 28-day carbonated samples, respectively.

**Figure 3:**
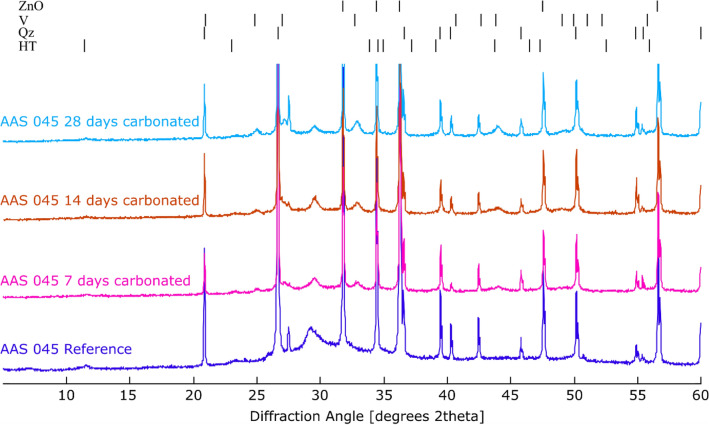
XRD diffractograms of specimen AAS 045 in a reference state and after each stage of carbonation. *ZnO* zincite internal standard, *V* vaterite, *Qz* quartz, *HT* hydrotalcite.

The carbonation of the alkali-activated slag specimens results in the formation of carbonate minerals and a reduction of the amorphous content (due to reaction with CO_2_). As is often the case for accelerated carbonation experiments^6,24,50^, the metastable vaterite formed instead of its stable polymorph calcite. The amount of vaterite formed depends on both the water to binder ratio of the mixture and the duration of carbonation as shown in Fig. 3 and Table 3. A longer carbonation duration resulted in a higher vaterite content as observed for AAS 045. The vaterite content after 14 days of carbonation is not much higher than after 7 days. However, this does not mean the carbonation degree of these two carbonation periods is similar because amorphous calcium carbonate can be formed from 7 to 14 days of carbonation as proved later by FTIR results. In AAS 035, the carbonation is markedly slower: after 28 days of carbonation only small amounts of vaterite (1.2%) could be observed in the XRD diffractograms. Overall, the amount of vaterite formed after 28 days of carbonation is the highest in AAS 055 (13.4%), indicating that a higher w/b ratio induces a higher carbonation rate due to a faster diffusion of CO_2_^51^.

### Fourier-transform infrared spectroscopy

**Figure 4:**
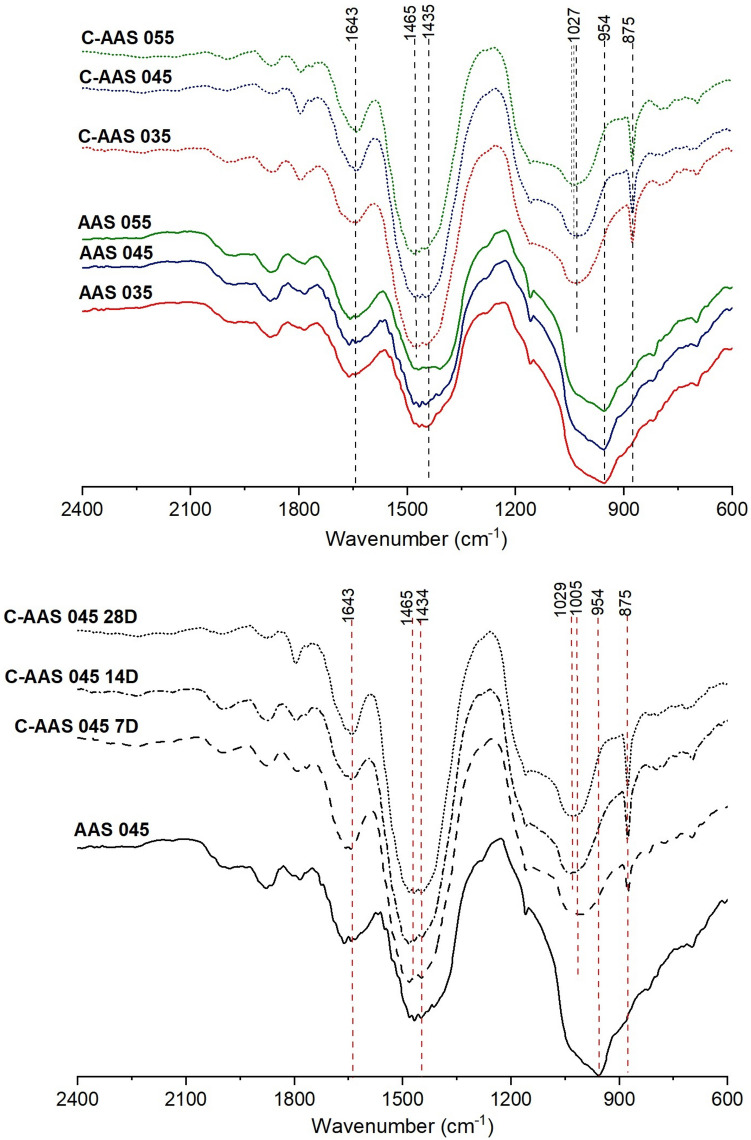
FTIR spectra of uncarbonated (AAS), 28-day carbonated alkali activated slag (C-AAS) at different w/b ratios (top) and C-AAS with w/b ratio of 0.45 (C-AAS 045) at 7, 14 and 28 days of carbonation (bottom).

In uncarbonated samples (Fig. 4 (top)), a band at 1643 cm^−1^ is detected, related to the bending vibration modes of H–OH bonds as chemically bound water in activated slag^52^. The band ranging from 1500 to 1400 cm^−1^ relates to the asymmetric stretching mode of C–O bonds of carbonate ions, which could be from the CaCO_3_ in AAS or the occurrence of natural carbonation during sample preparation as the powdered state is vulnerable to be carbonated. However, the latter may be ignored because samples were controlled to avoid exposure to atmospheric CO_2_. The asymmetric stretching vibration of Si–O–T bonds (where T is Si or Al) is observed at 954 cm^−1^, representative for the environment of SiO_4_ (silicate sites) in the C-A-S-H gels^38^. The increase in w/b ratio does not significantly influence the AAS structure as this band is similar for all samples with various w/b ratios. Only a slight difference can be observed in AAS 055, which shows a broader band at 1500–1400 cm^−1^ and a slightly lower intensity of Si–O–T, probably suggesting a lower degree of geopolymerization in AAS 055.

After accelerated carbonation, in the region from 1500 to 1400 cm^−1^, the right shoulder at approximately 1400 cm^−1^ diminishes, and the left shoulder gravitated toward 1500 cm^−1^ becomes larger. Comparing to the FTIR characteristic frequencies of Ca-carbonates from the report of Andersen^53^, a broader band is found in this region (1500 to 1400 cm^−1^), which suggests the presence of amorphous CaCO_3_. Furthermore, higher wavenumbers in carbonated AAS samples indicate the appearance of vaterite or aragonite. The sharp peak at 875 cm^−1^ can be assigned to vaterite. Based on these signals and the XRD detection, it is assumed that vaterite is the predominant Ca-carbonate product after 28-day carbonation. Furthermore, signals of Na-carbonates are not identified in FTIR spectra, in agreement with XRD results. In the range between 1200 and 900 cm^−1^, a remarkable shift of the main Si–O–T vibration band from 954 to around 1027 cm^−1^ is observed, indicating a higher degree of geopolymerization of silicates after carbonation. Li et al*.*^54^ and Zhang et al*.*^21^ also reported bands at high wavenumbers similar to those detected in the carbonated samples of this study.

Amongst the three carbonated samples, a specific difference can be found at the main Si–O–T bands, which shift toward a higher wavenumber for samples with higher w/b ratios from around 954 cm^−1^ to 1027, 1035 and 1041 cm^−1^ corresponding to the w/b ratios of 0.35, 0.45, and 0.55, respectively. This suggests that carbonated samples with higher w/b ratio are more cross-linked, and probably polymerized further under carbonation. A higher decalcification of C-A-S-H gels is also expected in these cases. In addition, the degree of polymerization also depends on the duration of carbonation, as shown in the Fig. 4 (bottom) by the shifting in the spectra of carbonated AAS 045 after 7, 14, and 28 days of carbonation. The centered peaks of the main Si–O–T bands shift to higher wavenumbers with ongoing carbonation, which indicates an increase in crosslinks in the gels during carbonation. In agreement with XRD results, the intensity of vaterite peaks at 875 cm^−1^ also increases, especially in the first 14 days of carbonation, indicating that the carbonation rate is accelerated in this period.

In order to examine the changes in carbonate products and chemical arrangement in detail, several studies^21,55^ deconvoluted FTIR spectra focusing on the main band of 1300–800 cm^−1^. They suggested that the appearance of Q^1^, Q^2^, Q^3^, and Q^4^ sites corresponds to the signals at 865, 898–1000, 1088, and 1136 cm^−1^, respectively. In this study, it is assumed that AAS samples contain mostly Q^1^ and Q^2^, while Q^3^ and Q^4^ are preferred in carbonated AAS samples. However, the complex environment around Si centers in AAS structures considerably affects the signals of Si–O vibrations. In that sense, the combination with NMR analyses could result in a better quantification of the C-A-S-H structure compared to the use of FTIR analyses alone.

### Solid-state MAS NMR results

#### ^27^Al MAS NMR

**Figure 5:**
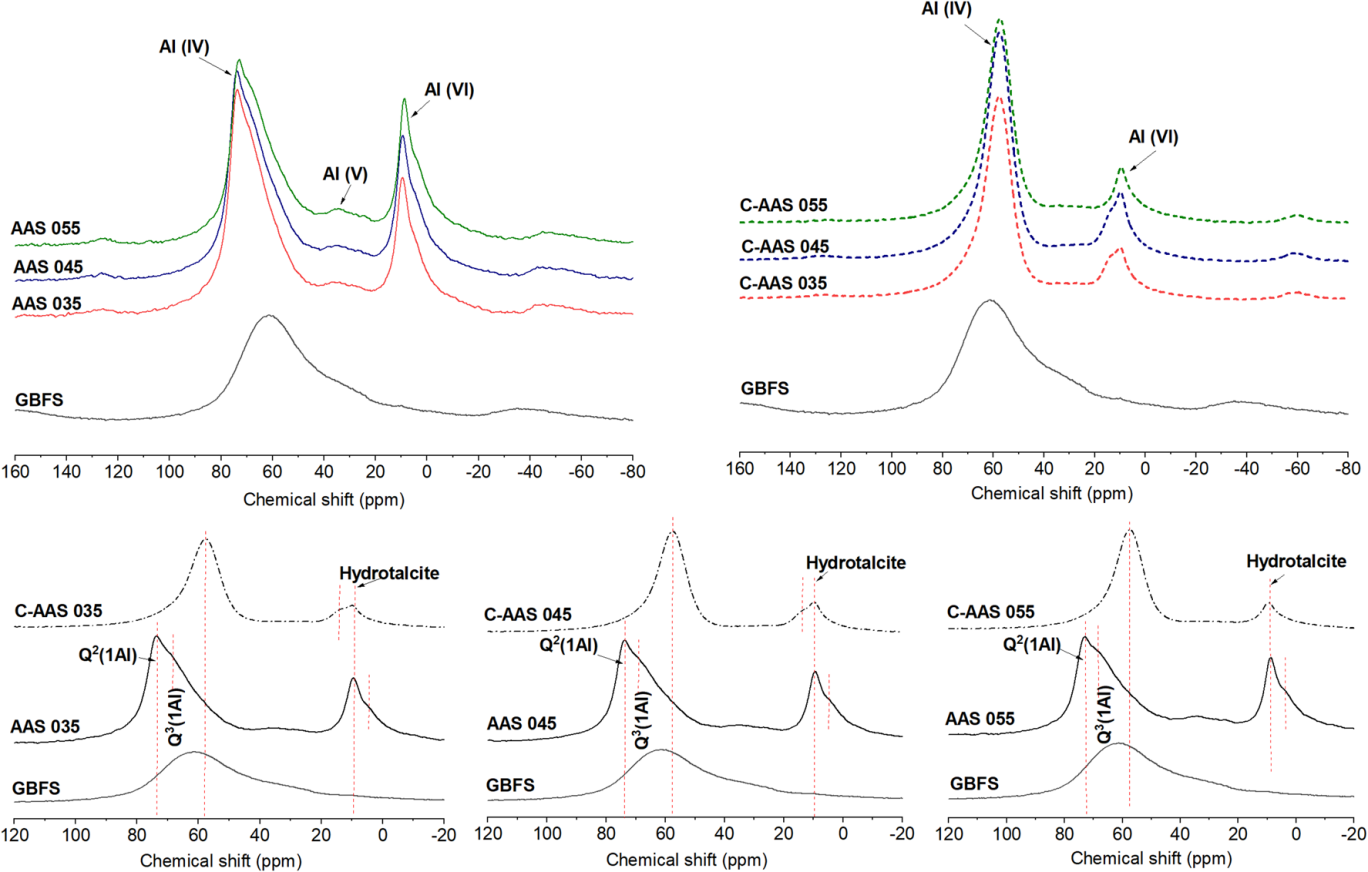
^27^Al MAS NMR spectra of anhydrous slag (GBFS), uncarbonated alkali activated slags at 28 days of curing (AAS), and carbonated alkali activated slag after 28 days of carbonation with 1% CO_2_ and 60% RH (C-AAS) at different water to binder ratios.

Figure 5 shows the ^27^Al MAS NMR spectra of AAS samples before and after carbonation. The spectrum of the raw slag shows a broad resonance ranging from 10 to 80 ppm, demonstrating the high disorder of the slag precursor as also indicated by the typical amorphous hump in the XRD pattern (Fig. 2). The centered resonance at around 67 ppm is assigned to the tetrahedral Al environments, which indicates that Al exists in the raw material mainly under fourfold coordination, Al(IV). Upon alkaline activation, the reaction products are recognized by two predominant regions centered at ~ 73 ppm, ~ 10 ppm, and a very minor contribution around 37 ppm, which correspond to the tetrahedral aluminum Al(IV), octahedral aluminum Al(VI), and pentahedral aluminum Al(V) environments, respectively^46^. Comparing to the spectrum of anhydrous slag, a higher intensity and narrower shape of the Al(IV) resonance is found, suggesting that Al(IV) becomes incorporated in C-A-S-H phase under bridging tetrahedral Al^47,56^. This Al(IV) region shows two resonances: one at 73 ppm and one at around 68 ppm appearing as a shoulder, which are supposed to be Q^2^(1Al) and Q^3^(1Al) sites^28,47^, respectively. These sites are charge-balanced with H^+^, Na^+^ and with more positive charges such as Ca^2+^ ions. The octahedral Al location with a peak centered at 10 ppm and a small shoulder at 4 ppm is assigned to layered double hydroxide phases^56^ (LDHs) with mainly hydrotalcite (Mg–Al), which agrees with the detected signal of hydrotalcite in the XRD results. Especially, these peaks are present in both uncarbonated and carbonated samples, meaning that, even if hydrotalcite is carbonated, i.e. for sample AAS 0.45 as XRD results indicated a relative hydrotalcite decrease, it is not fully carbonated^47,57^, which is confirmed more conclusively in the ^29^Si MAS NMR. In addition, the signal of Al(V), shown by a minor intense peak around 37 ppm is observed, which represents the remnant unreacted slag in the materials^57^.

Upon 28-day exposure to CO_2_, the specimens witness a shift of the Al(IV) region from around 73 to 58 ppm, which suggests a higher silicate content than oxhydryl surrounding Al center, leading to a lower electron density and thereby a lower aluminum chemical shift of this region^47^. Combined with the symmetry of this peak after carbonation, it is an indication that the higher the cross-linkage in the C-A-S-H phase is, the more homogeneous the Al sites in its structure are. A significant reduction of Al(VI) is also observed, while the resonance of Al(V) has mostly disappeared. That means the unreacted slag is transformed into aluminosilicate phases, leading to the dominance of only Al(IV) in C-AAS samples compared to both Al(IV) in C-A-S-H and Al(VI) in alumina-rich phase of AAS ones. This is also consistent with the ^29^Si MAS NMR results (discussed later) that clearly show a decrease in content of unreacted slag after carbonation. Besides, a very small shoulder at 14 ppm is also detected on C-AAS 035 and C-AAS 045. This signal is supposed to a “carbonated” LDH phase as a result of ion-exchange process during carbonation of hydrotalcite, or the existence of Ca, Al-LDH such as stratlingite^30^ in the system. The appearance of these phases may attribute to the reduction in the intensity of hydrotalcite at 10 ppm. However, AFm layers or even hydrotalcite types of LDHs are partially ordered forms in the C-A-S-H interlayers^28^, which make it difficult to be detected and distinguished clearly by XRD.

The water to binder ratio does not significantly influence the C-A-S-H structure of reference and carbonated AAS as similar characteristics amongst the samples are observed. However, if a relative comparison between the intensity of Al(IV) and Al(VI) is carried out, Al(VI) seems to be more pronounced with an increase of w/b ratio of uncarbonated AAS, while this is not observed in carbonated AAS. This suggests that the lower w/b ratio could allow the formation of more C-A-S-H phase. However, ^27^Al MAS NMR should be considered as a qualitative assessment rather than a quantitative evaluation^58^, and a quantitative deconvolution of ^29^Si MAS NMR will quantify these effects (see further).

#### ^23^Na MAS NMR

**Figure 6:**
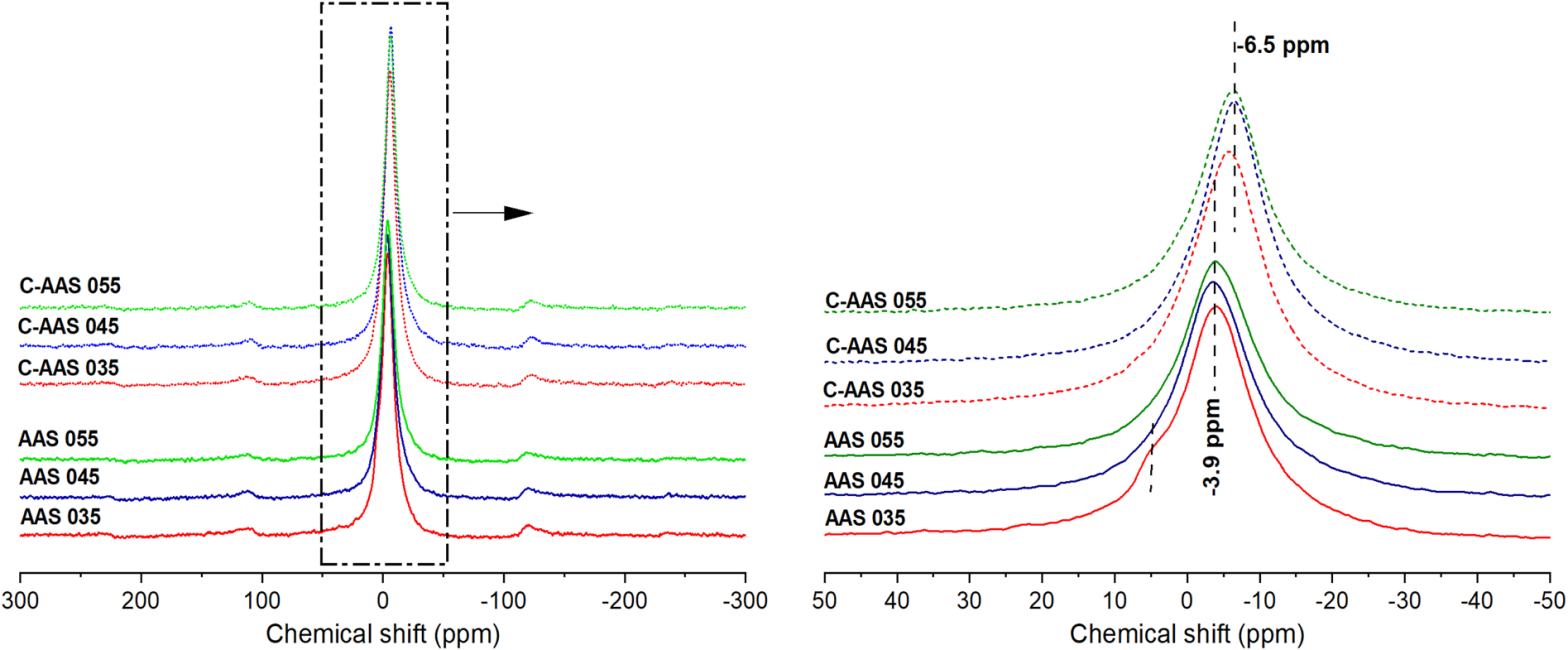
^23^Na MAS NMR spectra of uncarbonated alkali activated slags at 28 days of curing (AAS), and carbonated alkali activated slag after 28 days of carbonation with 1% CO_2_ and 60% RH (C-AAS) at different water to binder ratios.

The ^23^Na MAS NMR spectra of uncarbonated and carbonated AAS samples are shown in Fig. 6 by a single broad resonance. The resonance centered at − 3.9 ppm indicates Na in a coordination 6–7, which is associated with Na^+^ in a charge-balancing role in the C-(N)-A-S-H network^59^. Upon 28 days of carbonation, this resonance shifts toward a lower chemical shift at around − 6.5 ppm, showing an increase in the coordination number of Na^60^. This is in line with the results of ^27^Al MAS NMR and ^29^Si MAS NMR, which show that AAS is more cross-linked after carbonation. The trend of shifting in ^23^Na resonance is similar to that of Al(IV) in ^27^Al NMR spectra, which again suggests that the primary role of Na^+^ is to balance the net negative charge brought by Al to the Si–O–Al chain^61^. This is also consistent with XRD results, which show no Na-carbonate precipitation after carbonation. In addition, the similarity amongst the spectrum of samples with various w/b ratios before and after carbonation again illustrates that the role of w/b ratio is less important to the C-A-S-H structure. The only different characteristic that can be observed is that the shoulder around 5 ppm along with the main peak of the low w/b ratio sample (AAS 035) appeared with a less coordinated Na (smaller or equal to 5) beside a higher coordinated Na as the main Na state^60^. This indicates a variation of solvated Na^+^ in the hydration state in pore water of the specimens^46^. Therefore, the broader peak toward a higher chemical shift is evident to the low H_3_O^+^ in pore solution of AAS 035.

#### ^29^Si MAS NMR

**Figure 7:**
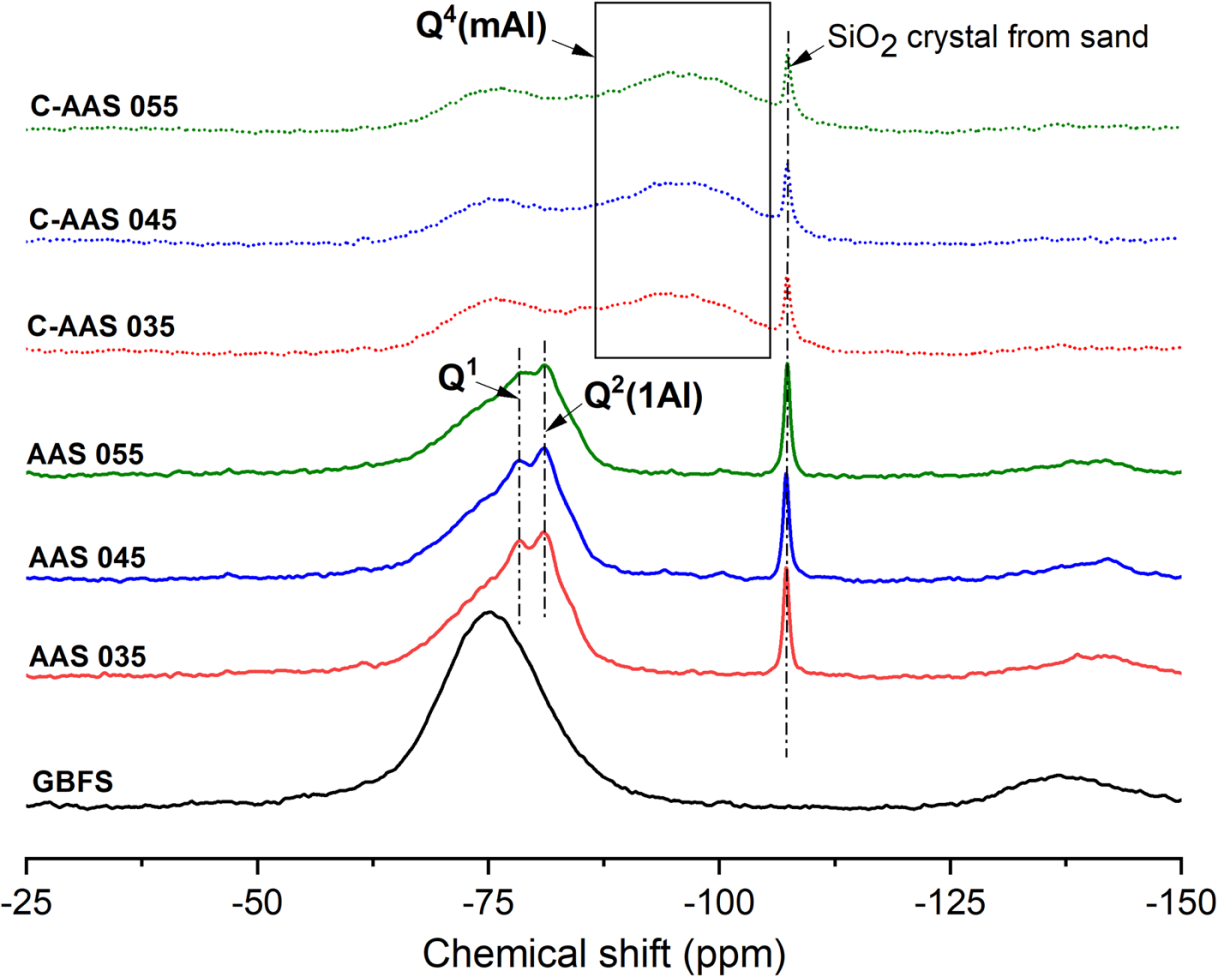
^29^Si MAS NMR spectra of anhydrous slag (BFS), uncarbonated alkali activated slags at 28 days of curing (AAS), and carbonated alkali activated slag after 28 days of carbonation with 1% CO_2_ and 60% RH (C-AAS) at different water to binder ratios.

Figure 7 shows the ^29^Si MAS NMR spectra of uncarbonated and 28-day carbonated AAS with different w/b ratios. In general, the C-A-S-H structures are not much different either for uncarbonated or carbonated AAS specimens with different w/b ratios, whereas there is a significant evolution of their structure (with the same w/b ratio) due to carbonation. The phases become more cross-linked witnessed by a shift from Q^1^ and Q^2^ sites to Q^4^ species. The resonance of GBFS is observed in all samples when comparing with the spectrum of slag precursor, suggesting the existence of remnant slag in both uncarbonated and carbonated AASs.

**Figure 8:**
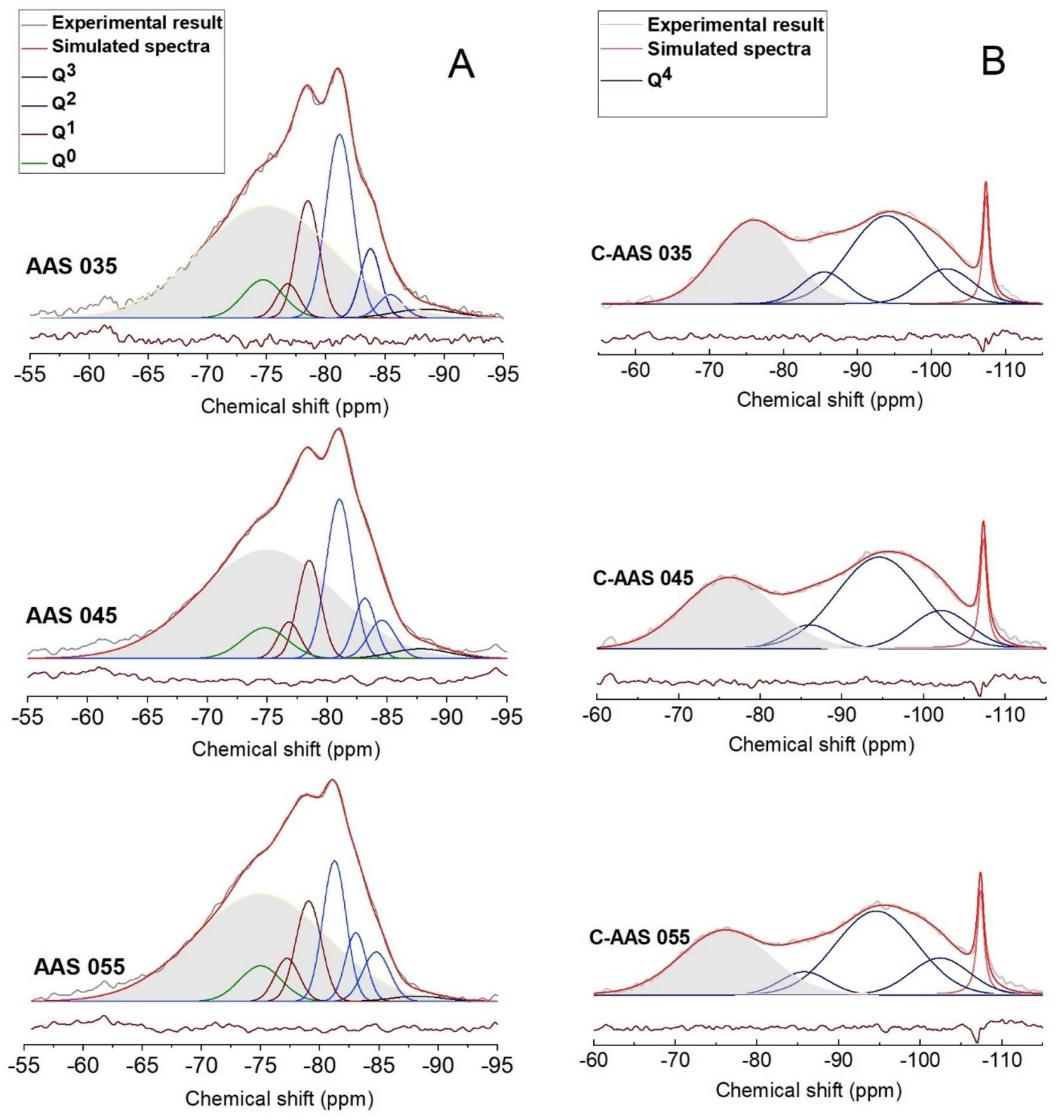
Deconvoluted ^29^Si MAS NMR spectra of uncarbonated alkali activated slags at 28 days of curing ((**A**) column), and carbonated alkali activated slag after 28 days of carbonation with 1% CO_2_ and 60% RH ((**B**) column) at different water to binder ratios.

To better understand the structure of samples via their ^29^Si MAS NMR spectra, each spectrum is deconvoluted, allowing a detailed comparison between component peaks, simulated spectrum and the experimental data as shown in Fig. 8. The broad region from approximately − 60 to − 95 ppm with a maximum at around − 75 ppm corresponds to the unreacted slag. In uncarbonated mortars, the peak residing at − 74.7 ppm can be assigned to Q^0^ species which encompass Si in isolated silica tetrahedral in the products^47^. The peaks located around − 78 ppm is identified as Q^1^, which represents to the chemical environment of Si at the end of a chain of silicate tetrahedral of C-(A)-S-H^62^. Herein, Q^1^ sites are represented by two peaks as Q^1a^ and Q^1b^ at − 76.8 and − 78.5 ppm, respectively, indicating the bonding effect of Q^1^ units with Ca^2+^, Na^+^ or H^+^ in the environment. This changes the chemical shift of Q^1^ sites^28^. Three types of Q^2^ groups are detected in these alkali activated products including Q^2^(1Al) sites assigned at around − 81 ppm, Q^2b^ and Q^2p^ sites at approximately − 84 ppm and − 85 ppm, respectively. These Q^2^ groups confer to the middle chain silicates of C-(A)-S-H phases^47,56^. Interestingly, the signals representative of the high cross-linked sites in gels as Q^3^ are not well observed although a very small peak of Q^3^(1Al) at around − 88 ppm is still detected. This is not in line with previous studies^28,47,56^. One explanation may be the lower activator modulus (Ms) used in this current study (Ms = 0.45) as Gao et al*.*^47^ reported that an increase of Ms to 1.7 or higher can accelerate the alkali activation resulting in a higher number of Q^3^ groups. Comparing amongst the three AAS with different w/b ratios, it is found that the difference only comes from the quantity of silicate groups in C-(A)-S-H, implying that the w/b ratio does not define the types of Si groups formed in these phases.

After 28 days of accelerated carbonation, remarkable changes in the structure are noticed as shown in Fig. 8. After carbonation, the chain structure of C-(A)-S-H containing predominantly Q^1^ and Q^2^ silicate species shifts into a more cross-linked gel, evidenced by the presence of four-connected silicate units (Q^4^) in the deconvoluted spectra of carbonated AAS samples. Similar to uncarbonated AAS mortars, a difference in the spectra of three carbonated AAS corresponding to the three w/b ratios is not clearly observed, implying a less important role of w/b ratio in the formation of C-(A)-S-H structure. In all carbonated mortars, peaks at approximately − 86, − 94, − 102 ppm can be identified, accounting for the presence of Q^4^(4Al), Q^4^(3Al) and Q^4^(2Al) units in the gels^63^, respectively. This observation is consistent with the lower ppm range and high intensity of Al(IV) sites as indicated by ^27^Al MAS NMR, showing the formation of Al-rich gel.

The relative intensities of Q^*n*^ species (%) from deconvolution results are reported in Table 4. Interestingly, the unreacted GBFS in carbonated AAS is significantly reduced compared to the reference AAS, from around 55% to 36% in general, meaning that more than one third of unreacted GBFS has been transformed to C-A-S-H phase and/or carbonated products during carbonation. In particular, the Q^1^ and Q^2^ units in reference AASs are unidentified in carbonated ones and replaced by plenty of Q^4^ groups in which Q^4^(3Al) is dominant (over 40%), indicating the presence of an extremely high cross-linked carbonated aluminosilicate C-A-S-H. This alteration is supposed to be the result of the decalcification of C-A-S-H^28^. Notably, the absence of Q^1^ and Q^2^ groups as representative for reacted products of AAS suggests that both existing and newly formed C-A-S-H phases in the materials are fully carbonated during 28 days of carbonation. Regarding to the influence of the w/b ratio, there is only a slight difference in the relative intensity amongst Al-substituted silicate sites in AAS, similar to carbonated AAS. The largest difference is observed in samples with a w/b ratio of 0.35, evidenced by a higher percentage of unreacted slag after carbonation. This could be explained by a denser matrix formed before carbonation, which can diminish the diffusion of CO_2_ into the matrix for carbonation.

**Table 4:** Deconvolution of ^29^Si MAS NMR results of uncarbonated and carbonated alkali activated slags.

Samples	Site type										
	Unreacted GBFS (%)	Reaction products (%)									
		Q^0^	Q^1a^	Q^1b^	Q^2^ (1Al)	Q^2b^	Q^2p^	Q^3^ (1Al)	Q^4^ (4Al)	Q^4^ (3Al)	Q^4^ (2Al)
	− 75 ppm	− 75 ppm	− 77 ppm	− 78 ppm	− 81 ppm	− 84 ppm	− 85 ppm	− 88 ppm	− 86 ppm	− 94 ppm	− 102 ppm
AAS 035	54	6	3	10	18	5	2	2	–	–	–
AAS 045	56	5	3	9	16	5	4	2	–	–	–
AAS 055	55	6	4	10	13	6	5	1	–	–	–
C-AAS 035	39	–	–	–	–	–	–	–	9	41	12
C-AAS 045	36	–	–	–	–	–	–	–	7	44	13
C-AAS 055	36	–	–	–	–	–	–	–	7	44	13

The estimated uncertainty in site percentages is ± 1%.

### Mechanism of carbonation and its influence on AAS gel structure

The changes in gel structure of AAMs with various w/b ratios exposed to accelerated carbonation (1% CO_2_, 60% RH) have been thoroughly examined in this study, and it is revealed that calcium carbonate is the dominant crystalline carbonation product, in which vaterite is the main polymorph as observed by XRD. Most of the previous studies^16,28,54,64,65^ reported the formation of stable calcite as the major stable polymorph. However, those samples were carbonated under different conditions such as high relative humidity (more than 80%), with either a low CO_2_ concentration of 0.04% (natural carbonation) or high CO_2_ concentration (5%) but longtime exposure up to 3 years. In addition, early age curing was chosen (3 or 14 days) and some studies were performed on AAM powder. Those testing conditions could result in the transformation of CaCO_3_ polymorphs from the metastable to stable state. In this study, carbonation is performed under intermediate conditions of 1% CO_2_ and 60% RH on well-cured AAS specimens for a shorter period of 28 days, which could be the reason for the appearance of vaterite as the main crystalline carbonate. Also, no sodium crystalline phases is detected. This is in line with reported data^28^ as the formation of sodium carbonate is only expected under some specific carbonation conditions. Typically, the Na-rich carbonation product nahcolite can only be observed under a high CO_2_ concentration of 5% or higher^28^, which is much higher than the one applied in this study. The evidence of crystalline carbonation products is also indicated by FTIR spectra under characterized wavenumber signals of CO_3_^2−^, and particularly from ^23^Na MAS NMR, which provides a strong proof of the non-presence of Na-carbonate precipitates because Na is only present in a charge-balancing role for the C-(N)-A-S-H phase. Additionally, hydrotalcite is detected as a secondary product of AAS, which still remains after carbonation, proven by XRD and ^27^Al MAS NMR results.

Carbonation also strongly influences the amorphous aluminosilicate structure of AAS. Results of XRD quantification show the decrease of amorphous content during carbonation, resulting from the decalcification of C-(N)-A-S-H to form calcium carbonate precipitates. Particularly, the decalcification allows the aluminosilicate gel to become significantly cross-linked, as evidenced by FTIR and ^27^Al MAS NMR results. Furthermore, the deconvolution of ^29^Si MAS NMR spectra shows mainly Al-substituted Q^4^ species in aluminosilicate gel instead of Q^1^ and Q^2^ in uncarbonated gels, in line with data from previous studies^20,48^. However, it is worth noting that the content of unreacted slag in AAS specimens (around 55%) is significantly higher than reported data in literature^45,66^ with approximately 30%. The high amount of unreacted slag can be activated and then carbonated during the carbonation process. As a result, a lower unreacted slag content is observed in carbonated specimens compared to uncarbonated samples.

This study also clarifies the influence of the w/b ratio on the evolution of phases and the amorphous gel nanostructure in particularly. Overall, the w/b ratio does not influence the type of crystalline reaction products but slightly influences the structure of C-A-S-H, which becomes highly structurally ordered and better resistant to carbonation at a low w/b ratio (e.g. 0.35). Zhang et al*.*^38^ and Ismail et al*.*^67^ also found a small effect of the w/b ratio on the gel nanostructure of alkali activated materials based on blends of slag and fly ash with a w/b ratio in the range of 0.3–0.6. By using a combination of FTIR, ^23^Na, ^27^Al, ^29^Si MAS NMR, and especially deconvolution of ^29^Si MAS NMR to assess the gel structures of AASs, this study was able to provide a comprehensive picture on the effect of w/b ratio on the gel structure of AAS, which is typically characterized only by FTIR technique as normally seen in literature^38^.

**Figure 9:**
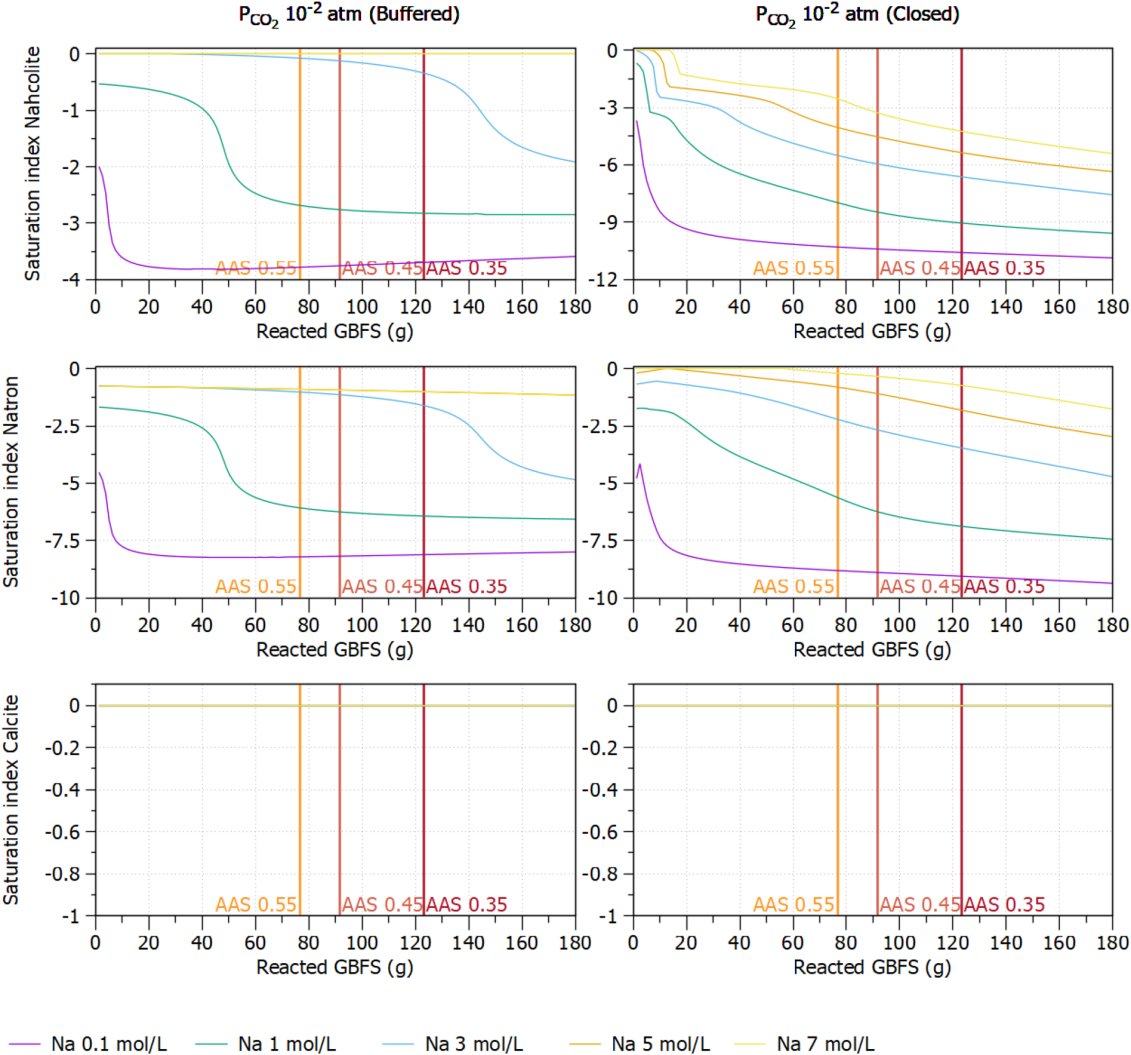
Saturation indices of nahcolite (top), natron (middle) and calcite (botom) in 1 l of water (20 °C) for 5 different Na concentrations. Vertical dashed lines indicate the amount of reacted GBFS determined by ^29^Si MAS NMR for 3 w/b ratios.

Based on the experimental evidence obtained from multiple characterization techniques, we propose a comprehensive mechanism for a diffusion-driven carbonation process of AAS as follows:

- Diffusion and dissolution of CO_2_ gas in the pore solution of AAS: This step liberates bicarbonate ($\text{HCO}_{3}^{-}$) and carbonate ($\text{CO}_{3}^{2-}$) ions. However, the former is unstable under the highly alkaline conditions (pH > 12.5) in the pore solution and transforms/converts into carbonate ions ($\text{CO}_{3}^{2-}$). These dissolution and hydration processes of CO_2_ have been well described in literatures for both cementitious materials^68^ and alkali-activated slag^32,69^.
- Formation of Na-carbonate products: $\text{HCO}_{3}^{-}$ and $\text{CO}_{3}^{2-}$ can react with the abundant Na^+^ in pore solution to form sodium carbonates including Na_2_CO_3_ (natron) and NaHCO_3_ (nahcolite) precipitates as intermediate products^70^.
  1 $${{\text{CO}}_{3}^{2-}}+2 \text{NaOH} \to {{\text{Na}}_{2}} \text{CO}_{3} +{2{\text{OH}}^{-}},$$
  2 $${{\text{HCO}}_{3}^{-}} + \text{NaOH} \to {{\text{NaHCO}}_{3}} +\text{OH}^{-},$$
  In order to verify whether natron and nahcolite are stable under our experimental conditions, we have performed a geochemical modelling to examine the saturation indices of nahcolite, natron and calcite. The reaction progress of GBFS was simulated assuming the release of Al, Ca, Na and Si from GFBS (other oxides were not included in the simulation) in 0.1 l of water (20 °C) with a given concentration of sodium (expressed as mol/l of Na) and a given pCO_2_ of 10^−2^ atm (i.e. 1% CO_2_ as used in the carbonation experiments) in a closed (i.e. initial 1% CO_2_) or open (buffered by maintaining 1% CO_2_) system. Furthermore, the effect of CO_2_ partial pressure is investigated by a variation of pCO_2_ in range 10^−2^–10^−1^ atm (i.e. 0.1 to 10% CO_2_ concentration). The reaction degree of GBFS determined by ^29^Si MAS NMR (Table 4), which is then used to calculate the total amount of Si, Ca, Al in 0.1 liter of water. The amount of activating solution is used to calculate the amount of Na and additional Si provided for the system. Details of these calculations can be found in the Supplementary Information. Simulations are done with PHREEQC^71^ with the CEMDATA18.1 database^72^ and additional constants for nahcolite and natron obtained from the BRGM Thermodem database^73^. The C-N-A-S-H solid solution model for alkali-activated model as reported in Ke et al.^30^ was used together with calcite, nahcolite and natron.
  As shown in Fig. 9 (top), in the system buffered with 1% CO_2_, which is close to conditions at the reactive surface, natron tends to be dissolved (negative saturation index) even at a very high Na concentrations of 7 mol/l in the pore solution. Nahcolite is only stable if the Na concentration is higher than 5 mol/l, which is much higher than the typical Na concentration in pore solution of hardened AAS (1–2 mol/l)^74,75^. The initial Na concentrations calculated from the mix compositions are 3.5, 2.7, and 2.2 mol/l for AAS with w/b ratios of 0.35, 0.45, and 0.55, respectively. The Na concentration is significantly decreased during polymerization (due to the precipitation of Na containing phases)^74^ to reach the average Na concentration of 1–2 mol/l. In the closed system (similar to the conditions at the location far away from boundary), both nahcolite and natron tend to dissolve even at Na concentration higher than 7 mol/l. The model also indicates that the possibility to have natron and nahcolite present in the system is lower with the increase of w/b ratios.
- *Formation of Ca-carbonate products* The dissolution of natron and nahcolite in the presence of Ca^2+^ (from decalcification of C-A-S-H and GBFS) could induce further carbonation reactions to form a stable precipitate CaCO_3_ as illustrated in Eqs. (3) and (4). As shown in Fig. 9 (bottom), calcite is oversaturated for both closed and buffered systems. The reactions (3) and (4) also increase the alkalinity in the pore solution with the formation of NaOH. With the high alkalinity and an abundance of unreacted slag in the matrix, the geopolymerization can continue to form additional C-A-S-H phase, which may be then carbonated. In this study, because of the high amount of unreacted slag in AAS, it may continue dissolving to provide Ca^2+^ in solution to react with carbonate ions and form calcite. Furthermore, apart from the direct carbonation, the continuous dissolution of GBFS could result in a formation of new less polymerized C-A-S-H as an intermediate product before further carbonation.
  3 $${{\text{Na}}_{2}} {{\text{CO}}_{3}}+{\text{Ca}}^{2+} + 2{\text{OH}}^{-} \to {\text{CaCO}}_{3 (s)} +2{\text{NaOH}},$$
  4 $${{\text{NaHCO}}_{3}}+{{\text{Ca}}^{2+}}+2{{\text{OH}}^{-}}\to {\text{CaCO}}_{3 (s)} + {\text{NaOH}}+ {\text{H}}_{2 }{\text{O}}.$$

**Figure 10:**
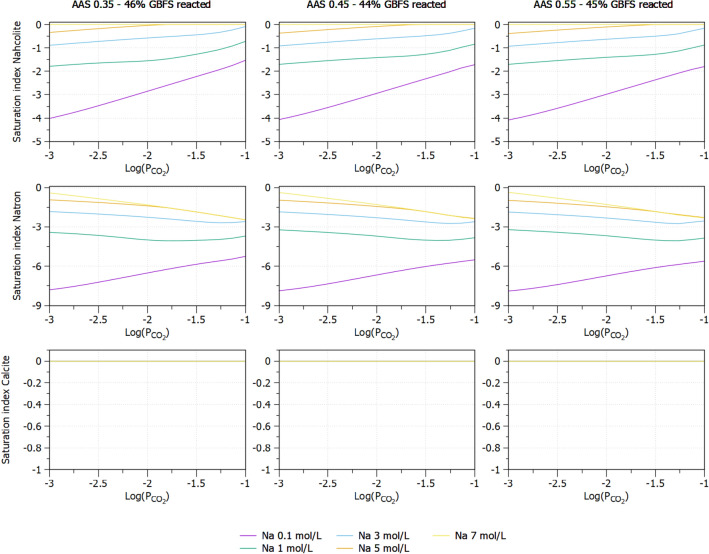
Effect of CO_2_ partial pressure on saturation indices of nahcolite (top), natron (middle) and calcite (bottom) of AAS with different w/b ratios.

The model also allows to investigate the effects of CO_2_ concentration on the formation of nahcolite, natron and calcium carbonate due to carbonation. It can be seen in the Fig. 10 (top) that the higher the CO_2_ concentration, the higher possibility the nahcolite formation. If the CO_2_ increases to 10%, nahcolite is mostly formed with Na concentration higher than 3 mol/l. Natron is unstable even with low CO_2_ concentration of 0.1%, while calcium carbonate is also formed regardless of Na and CO_2_ concentrations. Figure 10 also shows the limited effect of w/b ratio on the formation of carbonates.

## Conclusions and perspectives

The alteration of mineralogy and C-A-S-H structure of alkali activated slag under accelerated carbonation with 1% CO_2_, 20 °C and 60% RH were investigated in this work. The combination of multiple characterization techniques including XRD, FTIR and sophisticated solid state ^29^Si, ^27^Al, ^23^Na MAS NMR allows accessing insight into the evolution of the material’s structure at the nano scale. The results revealed that vaterite representative for calcium carbonate is the predominant carbonated crystalline phase of carbonated AAS, which results in a decrease in C-A-S-H content. No trace of sodium carbonate is found as commonly reported in literature. The FTIR and solid state MAS NMR in particular highlight an intensively cross-linked structure of the gel upon carbonation, in which Q^4^ species are dominant in the carbonated aluminosilicate gels instead of Q^1^ and Q^2^ sites in uncarbonated specimens. Furthermore, similar characteristics amongst samples with various w/b ratios suggest a limited role of w/b ratio on the structure of AAS before and after carbonation and the formation of carbonates. The w/b ratio only influences the formation of sub-components in the amorphous C-A-S-H phase without contributing to the formation of new crystalline and amorphous phases in both uncarbonated and carbonated AASs. It is worth noting that the w/b ratio may play an important role in the pore solution chemistry and microstructure (not investigated in this study), which are crucial in understanding carbonation mechanism, as discussed in the “Introduction” section.

Based on experimental observation and geochemical modelling, for the first time, we propose a carbonation process of AAS including (i) diffusion and dissolution of CO_2_; (ii) formation of immediate Na-carbonate products, which are not stable in Ca-rich solution due to C-A-S-H decalcification; and (iii) the formation of calcite as the stable CaCO_3_ polymorph, in which the alkalinity in the pore solution is maintained allowing a further geopolymerization to form additional C-A-S-H and/or further dissolution of slag to be carbonated later. This proposed process allows a better understanding of the carbonation mechanism of AAS and to explain well the absence of sodium carbonates and the reduction of unreacted slag under carbonation as experimentally observed.

## Supplementary Information

Supplementary Information.

## Data Availability

The raw datasets generated during and/or analysed during the current study are available from the corresponding authors on request.
